# Animal model considerations for chordoma research: reproducing the tumor microenvironment *in vivo* with humanized mice

**DOI:** 10.3389/fonc.2024.1330254

**Published:** 2024-03-13

**Authors:** Beatrice Campilan, Christian Schroeder, Emilija Sagaityte, Jonathan Arditi, Owen P. Leary, Ziya L. Gokaslan, Patricia L. Zadnik Sullivan, Margot Martinez-Moreno

**Affiliations:** Department of Neurosurgery, Rhode Island Hospital, Warren Alpert Medical School of Brown University, Providence, RI, United States

**Keywords:** chordoma, humanized mice, immunocompromised mice, immunotherapy, tumor microenvironment

## Abstract

Animal models have been commonly used in immunotherapy research to study the cell response to external agents and to assess the effectiveness and safety of new therapies. Over the past few decades, immunocompromised (also called immunodeficient) mice allowed researchers to grow human tumor cells without the impact of the host’s immune system. However, while this model is very valuable to understand the tumor biology and to understand the underlying mechanism of immunotherapy, the results may not always directly translate to humans. The tumor microenvironment has significant implications for tumor engraftment, growth, invasion, etc., and the immune system plays a critical role in shaping the tumor microenvironment. Human immunocompetent mice, also named humanized mice, are engineered mice that possess functional human immune cells. This *in vivo* model can be used to effectively study the effect of the human immune system to a human implanted tumor. Moreover, this can effectively mimic the response to treatment. This section is an overview of the current understanding of the different humanized mice that could be utilized to mimic the tumor microenvironment in chordoma.

## Graphical Abstract

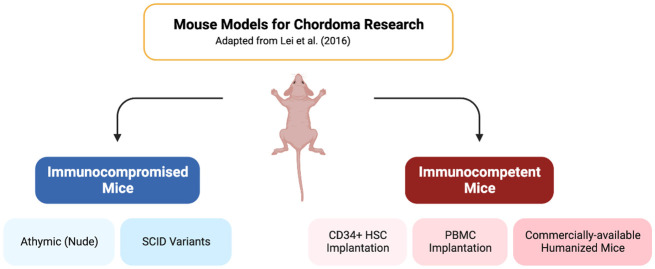

## Introduction

For decades, animals have served as invaluable tools for studying human biology in research. Among these, mice, in particular, have gained widespread use owing to their small size, ease of handling and maintenance, cost-effectiveness, rapid reproductive cycles, and their shared genomic, anatomical, and physiological characteristics with humans. As a result, they have made an indelible mark on biomedical research and have emerged as the primary model for investigating human diseases and conducting preclinical trials, thanks to the discipline of comparative medicine (1). With the expansion of our understanding of human diseases, there is an escalating demand for increasingly sophisticated mouse models. These models are not only essential for comprehending the biology of the diseases but also for evaluating potential therapeutic interventions. In this review, we will provide a concise overview of the animal models employed in cancer research, with a particular focus on chordoma. Additionally, we will examine the importance of utilizing a specific mouse model, humanized mice, in chordoma studies.

## Methods

### Search strategy

A literature review was performed using PubMed. This review did not receive funding, a full review protocol is available by request. Search terms included [(chordoma) AND ((mouse) OR (microenvironment))] on all literature published before August 16, 2023. Articles were independently screened by (C.S, B.C, M.M.M, O.L, J.A), full text of remaining studies were screened by (C.S, M.M.M).

Inclusion criteria included all English language papers describing novel immunocompromised mouse models in chordoma. Fifty papers were excluded, 32 did not include mice, 1 was a duplicate study, 3 were not in English, 4 mouse studies contained non-immunocompromised mice, 8 contained non-novel models, and 3 did not use chordoma as the model. The initial search yielded 92 unique articles with 42 papers remaining after inclusion criteria was applied.

## Chordoma

Chordomas are primary tumors of the axial skeleton and skull base, believed to be derived from primitive notochordal cells. The incidence of chordoma is approximately 0.08/100,000 (2). The progression of the tumor is slow and insidious, most often presenting clinically at the median age of 59 (3). However, the tumor can present in pediatric patients, most often presenting at age 10 (4). Since chordomas may present in the clivus, mobile spine, and sacrococcygeal region, symptoms can vary greatly. Clival chordomas typically manifest as cranial nerve palsy and headaches (5). While a mobile spine lesion typically presents with back pain in addition to neurological symptoms associated with the affected nerve roots (6). Sacrococcygeal lesions present with similar symptoms to mobile spine lesions with the addition of bowel and bladder complications (7). Current treatment for chordoma recommends radical en bloc resection with adjuvant radiotherapy. En bloc resection is recommended since the complete resection of the tumor with a wide margin and without tumor capsule invasion prevents the seeding of cancer cells, thus preventing recurrence (8). In addition to en bloc resection, some patients will receive adjuvant ration as it has been shown to improve disease-free survival, however, this is still debated (9). Currently, there are no medical treatments approved for chordoma (10). Creating a medical treatment could have utility in shrinking tumors preoperatively to reduce surgical margins and improve quality of life, as well as applied postoperatively to prevent recurrence.

The use of animals, particularly mice, in cancer research serves as an invaluable tool for advancing our understanding of various malignancies, including chordoma. The organism physiology and the tumor microenvironment is essential to study cancer and for conducting therapy testing: animals, with their biological complexity, provide a comprehensive perspective that cannot be replicated in *in vitro* models. This mini-review conducts a broad examination of the general use of mice in cancer research, with a subsequent in-depth exploration of its use in chordoma research, focusing on the use of humanized mice.

## Immunocompromised mice and it use in cancer research in general and chordoma research in particular

### Introduction to immunocompromised mice

Animal models have become indispensable tools in cancer and immunotherapy research, providing insights into tumorigenesis, the safety and efficacy of developing therapeutics, and the tumor microenvironment in which their interactions occur. Pre-clinical *in vivo* studies using these models have been key in the advancement from simple mass surgical resection to more specific target-based treatments in modern medicine (11). The use of murine models, in particular, has been well-established in the existing literature. Compared to other available models, mice are not only low in cost and easy to maintain but also have a short lifespan and reproductive cycle, exhibit high tumor growth rates, and are susceptible to genetic manipulation (12). Numerous murine cancer models have been developed since the previous century, each with its own advantages and limitations, and have contributed to the discovery of novel cancer control and prevention strategies. Immunocompromised mouse models are among the most frequently utilized approaches to evaluating tumor biology. Also referred to as immunodeficient mice, given their suppression or lack of a functional immune system, these specialized models have enabled researchers to elucidate the underlying mechanisms driving tumor cell growth without the confounding effects of immune response or rejection. Since their initial discovery in the 1960s, multiple strains of immunocompromised mice with varying degrees of genetic defects have been developed and commonly employed in chemotherapeutic/cytotoxicity studies and patient-derived xenograft (PDX) models (13).

Athymic nude mice were the first known immunocompromised model, discovered in 1962 by Grist (Ruchill Hospital, Glasgow, UK) and was first published by Flanagan in 1966 (14). Widely recognized for their lack of body fur (therefore the *nude* nickname), they also possess severe T-cell dysfunction due to abnormal thymus development; however, their innate immune system and B cells remain intact. Alternatively, severe combined immunodeficiency (SCID) mice are deprived of DNA-dependent protein kinase encoded by the deleted PRKDC gene, resulting in a shortage of both functional B and T lymphocytes. These were later the first immunodeficient mice to receive human hematopoietic stem cell (HSCs) and peripheral blood mononuclear cell (PBMCs) transplantation to generate the first humanized mice, as we explain below (15). To improve the engraftment efficiency of human tumors in these models, SCID/Beige mice were later established by crossbreeding SCID and Beige mice (16). While improvements were observed, it still left much to be desired.

Non-obese diabetic (NOD) mice were then identified in 1980, and their crossbreeding with SCID mice produced the NOD/SCID model. NOD/SCID mice lose functional NK cells in addition to T and B lymphocytes, though residual NK cell activity remains prevalent, limiting engraftment efficiency (17). To address this, several NOD/SCID-based immunocompromised derivatives with even further reduced or complete loss of NK cells were also established, including NSB, which lack β2 microglobulin, and NOJ, which are Jak-3 deficient (17–19).

**Figure 1:**
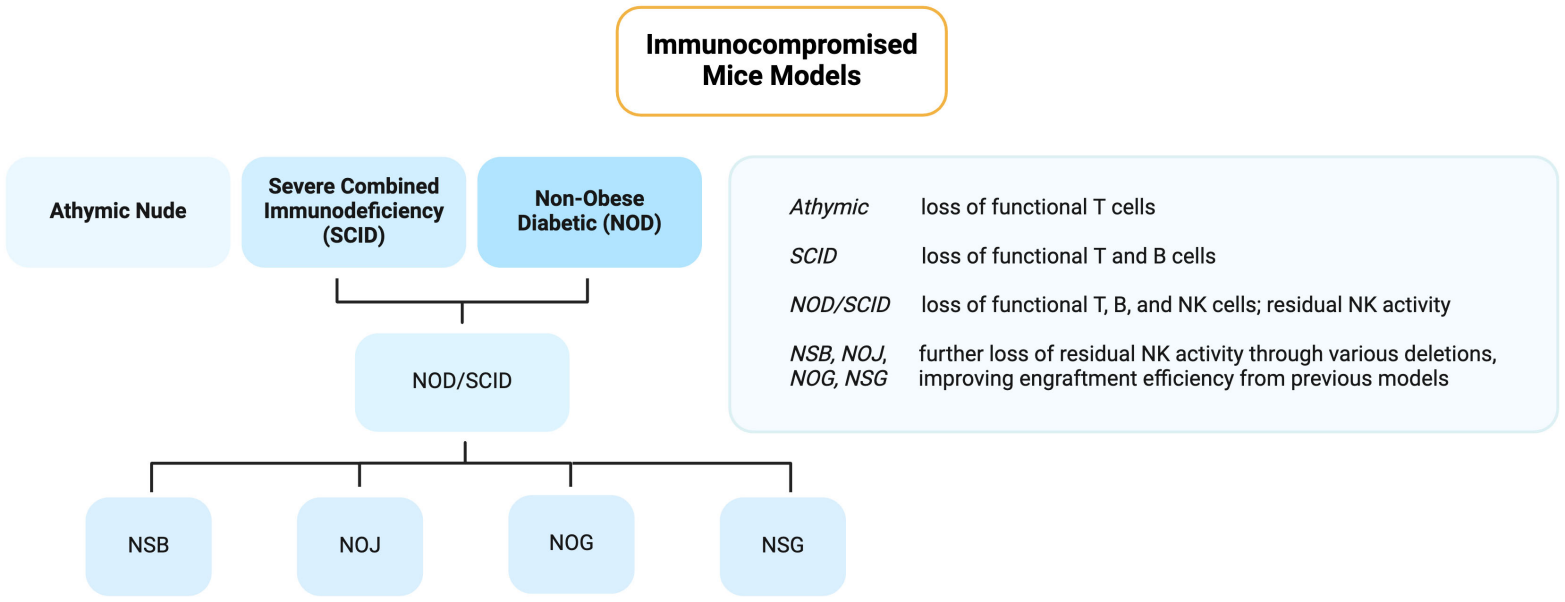
Flowchart of the different immunocompromised mice models used for cancer research. Created with BioRender.com.

In the early 2000s, Ito et al. (20) and Shultz et al. (21) developed two more NOD/SCID-related models: NOG and NSG, respectively. These are frequently utilized given their lack of mature lymphocytes and NK cells, leading to high engraftment of human cells and tissues (22). These features arise from the deletion of a functional common interleukin-2 γ-chain (NOD/SCID IL- 2rγ^null^), which is vital for the high affinity binding and signaling of IL-2, IL-4, IL-7, IL-9, IL-15, and IL-21. Without this molecule, NK development is halted. Their ability to survive beyond 16 months is therefore of particular interest in light of their severe impairments in innate immunity. Although both may also be referred to as NOD/SCID IL- 2rγ^null^, NOG possesses a partial deficiency of IL2rγ while NSG has a complete deficiency (17). These numerous NOD/SCID-related strains emphasize the range of immunodeficiencies available to researchers depending on their particular targets of interest, lifespan requirements, or reported engraftment rates. Since the generation of humanized mice bearing the interleukin 2 receptor gamma chain mutation (IL-2rγnull)^2^ in the change of the millennia, investigators have been able to successfully engraft human tissues, including HSCs, as we will delve into in more detail below. A summary of the immunocompromised mice models is graphed in **Figure 1**.

### Immunocompromised mice and its use in cancer research

It has been demonstrated that such immunocompromised mice models have served a pivotal role in understanding various cancers. For instance, both SCID and NOD/SCID mice were deemed a vital step forward in the study of acute myeloid leukemia (AML) (23). Relative to athymic nude mice, the SCID model led to improved engraftment rates of primary AML when injected intraperitoneally or implanted under the kidney capsules, and was found to be a reproducible system for the expansion of blastic human myeloid leukemias (24). NOD/SCID mice, with reduced NK cell and macrophage activity, produced superior engraftment rates and seemed to mostly preserve the morphologic and phenotypic characteristics of AML (25). Agliano et al. (18) have also systematically compared the transplantation of primary AML cells into NOD/SCID, NSB, and NSG strains and highlighted the faster engraftment observed in the latter. Other models have been similarly helpful in exploring head and neck squamous cell carcinoma, pancreatic cancer, non-small cell lung cancer, and prostate cancer, to name a few (26–29).

At present, the primary concern surrounding the use of immunocompromised mice is their limited replicability from preclinical to human trials. These models may have exceeded their capabilities as they are unable to faithfully represent the complex tumor microenvironment that is necessary for an accurate evaluation of immunotherapeutic approaches in humans (30). Nevertheless, they have been and continue to be instrumental in discerning disease pathology and serve as a bridge from bench to bedside therapies.

### Immunocompromised mice in chordoma research

Mouse models currently exist for chordoma research utilizing immunocompromised mice. Chordoma mouse models began with three different cell lines, each capable of forming a tumor after being implanted in immunocompromised mice (31–33). However, the first primary chordoma patient derived xenograft mouse model transplanted directly from a human patient was established in 2012 (34). Eventually this model of xenograft was modified to enable serial transplantation of primary chordoma tumors in mice (35). Athymic nude mice are the basis of many tumor grafting experiments due to the decreased likelihood of tumor rejection. This makes them a common model for chordoma xenografting as well through subcutaneous injection to the flank of cells combined with Matrigel (36).

A common specific mouse model used for cellular derived xenograft (CDX) and patient derived xenograft (PDX) in chordoma are BALB/C nude mice. These are athymic mice which are used as xenograft recipients through a subcutaneous injection of cells combined with Matrigel into either the hindleg, thigh, forearm, flank, and inguinal fold, all with success (37–46).

Another popular method for PDX and CDX in chordoma are SCID variants of mice, differing from the most rudimentary model of SCID to the most modern NOD-SCID interleukin 2 receptor gamma (NSG) null mice (33, 47–57). These models follow suit with other studies, injecting the cells into various locations subcutaneously. The exception to this only study that implanted the cells into the subscapular fat pad of the mice (58).

Other models of PDX chordoma in SCID mice have also been done to try to emulate the growth of clival chordomas. For example, one model implants the cells subcutaneously in the epicranial space, creating an anatomical environment closer to the tumor in humans (59). Other models exist that use similar models but are less commonly seen in chordoma studies. Such as the use of athymic CD-1 *nu/nu* mice (60). Nearly every model uses subcutaneous injections of cells into mice. However, one student implanted chordoma cells into scraped areas of the sacrum in addition to doing subcutaneous flank injections (61). This method yielded a statistically significant 23% increase in engraftment success in the scraped sacrum compared to the commonly used flank.

While these models are excellent for studying chordoma *in vivo*, they lack the capacity to study the tumor microenvironment created in an immunocompetent human host. The tumor microenvironment has been shown to be critical in understanding the progression of chordoma and outcomes in patients (62, 63).

## Humanized mice

### Introduction and definitions

In recent years, there has been a significant advancement in our comprehension of the interactions between oncogenesis and the host’s immune system. Concurrently, there has been remarkable progress in the development of therapeutics aimed at augmenting the immune system’s anti-tumoral responses. Notably, immune checkpoint blockade therapy, exemplified by Keytruda^®^ (pembrolizumab), an FDA-approved humanized monoclonal antibody that binds to the PD-1 receptor on T-cells, preventing tumor cells from suppressing the immune response (64, 65) has emerged as one of the most successful treatments available to the public. As a result, there is a growing demand for a model that can faithfully replicate the human immune system to evaluate new therapies. One such model is the humanized mice.

In broad terms, humanized mice refer to mice that have been generated or bioengineered by incorporating human cells or tissues into the mouse model. This concept extends beyond models of immunity and encompasses various types of cells, tissues, or molecular components from the human system. For instance, mice that are immunocompetent and express human genes through transgenesis are categorized as humanized mice. These mice can carry multiple transgenes, whether or not they are associated with the immune system. Over the past three decades, numerous scientific studies have utilized these models to introduce a variety of human genes, both within and outside the realm of immunology (66, 67), models that are very useful because they can inherit and express human genes (68). To clarify, the term “humanized/immunocompetent” as discussed refers to the use of immunocompromised mice that have been engrafted with functional human cells or tissues, resulting in the development of a functional human immune system within the mouse model. These mice have become increasingly important over the past decades as preclinical models of disease (69).

Immunocompetent or humanized have been used since 1988 when SCID mice were transplanted with human hematolymphoid organs: fetal bone, liver, thymus, spleen, intestine, skin, and lymph node (70). The breakthrough that allowed the study of HIV pathogenesis not only paved the way for research on infectious diseases but also unlocked various applications for humanized mice in other fields, including cancer (71). During the past 3 decades, diverse protocols have been presented to generate humanized mice (72). The mice that are most commonly used to create humanized mice are the SCID model-based (SCID, NOD, NSD or NSG) that, as explained above, its genetic disorder is characterized by the complete disability of the adaptive immune system to generate and sustain an immune response, due to the lack of B and T cells (13, 72–75). These mice can be stably engrafted since they lack this response.

### Types of humanized mice

In this context, humanized mice can be classified into two categories depending on the type of human immune or hematopoietic cells that are engrafted. Mice can be engrafted with human CD34+ HSCs, hCD34+, or they can be engrafted with human PBMCs, hPBMC.

#### hCD34+ mice

The hu-SRC mice, which stand for human SCID repopulating cell mice, are the type of immunocompromised mice that serve as recipients for human CD34+ HSCs. These mice produce T, B, and myeloid cells, especially Natural Killer (NK) cells, derived from the engrafted CD34+ HSCs. The tissue sources of CD34+ cells in this engraftment process are human bone marrow, cord blood, or mobilized peripheral blood. However, the percentage of CD34+ varies depending on the tissue source or the mobilization agent (76, 77). Importantly, these cells mature within the mouse host, leading to the development of tolerance to the host’s antigens. This unique characteristic allows researchers to utilize this model for studying not only the development of the hematopoietic lineage but also the mechanisms involved in immune system development and the generation of primary immune responses, particularly with naïve T cells (77). Moreover, the lifespan of the newly acquired human immune cells results in a 12-week window before exhaustion. This timeframe allows for the study of immune cell memory response and long-term antitumor effects (78). Furthermore, this model facilitates the study of drug immunogenicity as it possesses a complete human immune system (79).

#### hPBMC mice

The humanized hu-PBL mice, also known as human peripheral blood leukocyte mice (hPBMC), are NSG immunocompromised mice engrafted with a pool of human PBMCs, resulting in the creation of mouse chimeras (80). These mice are often considered transiently humanized mice since the mature T cells can last between a few weeks and, much less often, a few months (81). This is caused by the Graft-versus-host disease (GvHD), which is usually manifested at week 4 after engraftment. Since the engrafted cells are already matured, they end up rejecting the host tissues given the differences in the major histocompatibility complex (MHC) (82). The advantage lies in the cost-effectiveness of this model, albeit with a limitation on experiment duration. Experiments conducted within a concise timeframe, as seen in cancer research, specifically focus on T and NK cells, including investigations into immune checkpoints (83).

In both PBMC and HSC-engrafted mice models, human immune cells naturally develop, migrate, and infiltrate the tumor microenvironment, mirroring the conditions observed in primary patient tumors. This model is extremely useful for cancer research since it provides a model to study tumor-immune system interactions (13, 30, 69, 72, 74, 75, 84). An overview of these two types of humanized mice is summarized in **Table 1**.

**Table 1:** A comparison between the humanized mice models hPBMC and hCD34+. Created with BioRender.com

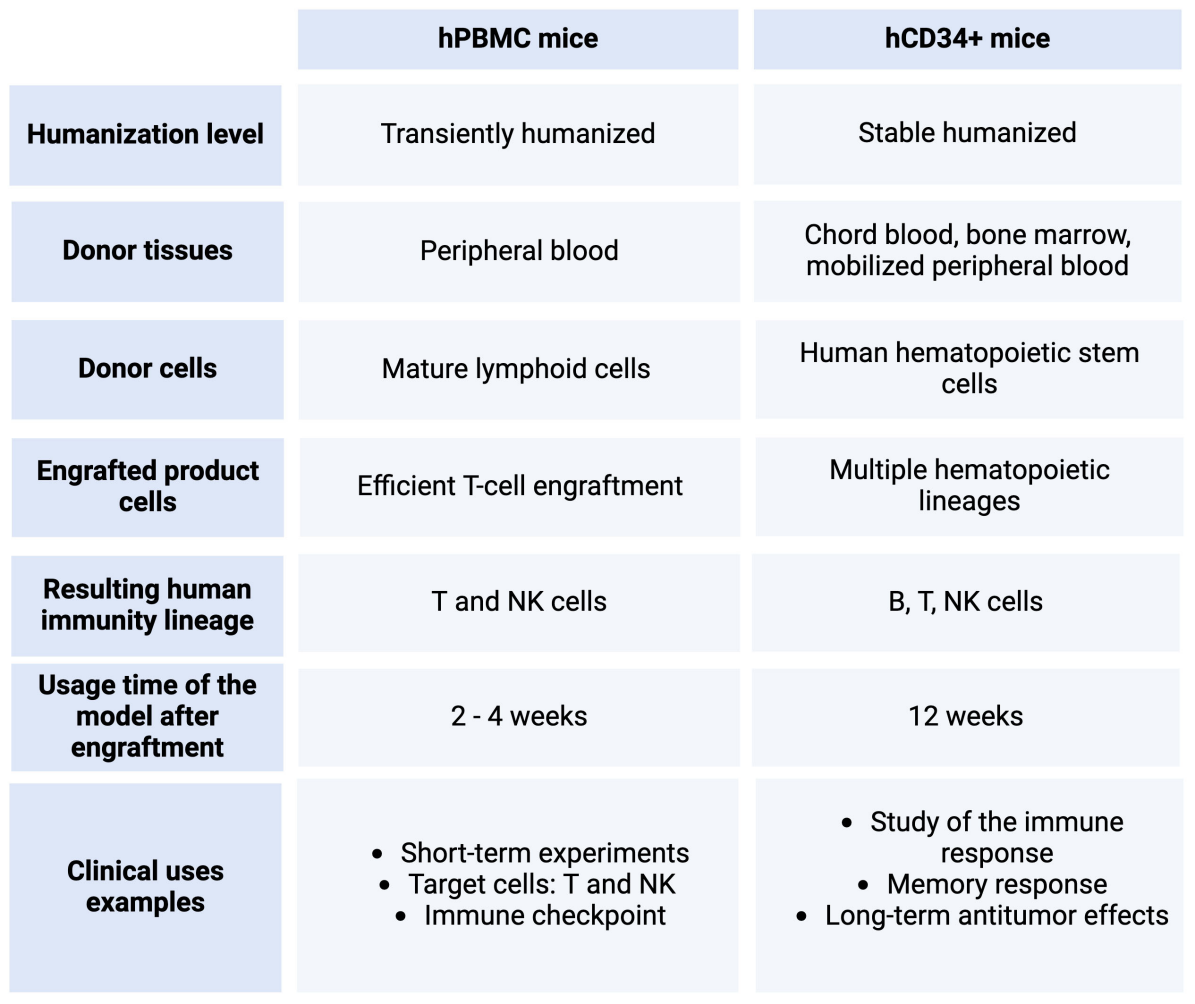

### Humanization protocols and commercially available mouse strains

Both immunological models of mice *humanization* can be performed on different immunocompromised strains, being the NSG™ (from Jackson Laboratories) and the NOG (from Tactonic) the most commonly used to create the humanized models.

As mentioned earlier, NSG mice, which are immunodeficient (NOD/SCID) mice with a specific mutation in the IL-2 receptor common gamma chain (IL2rγ ^null^), exhibit significantly enhanced engraftment capabilities. These mice are extensively employed in research related to immunity, infectious diseases, cancer biology, regenerative medicine, and various other fields (72, 74). The generation of commercially available cohorts of stem cell humanized mice starts by the treatment of the NSG/NOG mice with busulfan to clear the stem cell niches, alternative to the irradiation, first described at Tisdale’s laboratory (85). This approach enhances the engraftment of CD34+ human hematopoietic stem cells derived from cord blood, resulting in the development of chimeric human CD45+ (hCD45+) fully matured B and T lymphocytes within 4 to 5 months, all while minimizing associated toxicity (85–90). These engrafted cells differentiate into various immune cell lineages, resulting in a chimeric mouse possessing a human immune system. This system is characterized by hematopoietic stem cells reconstituting into CD4+ T helper cells and cytotoxic CD8+ T cells, along with reduced levels of B cells, macrophages, NK cells, and dendritic cells (89–91). Moreover, new transgenic strains that express human hematopoietic growth factors have conferred these mice with a more robust immune system and with the expression of human HLA molecules (92).

### The need and use of humanized mice models

The benefit of utilizing commercially available humanized mice is that the companies typically assess chimerism and engraftment levels in the animals before they are shipped. However, a drawback of this approach is that it may limit the experimental window, potentially reducing the time available for conducting experiments, as humanized mice suffer a shortened survival time and may have HSC exhaustion (93).

The use of humanized mice to study immunotherapies has served to the advancement of medical research, to reduce animal testing and to accelerate drug testing and development (69, 94, 95). For example, the immune checkpoint inhibitor that we started this section talking about, keytruda^®^, has been extensively used in humanized mice since its commercialization in 2013 in a wide array of cancers and in combination therapies (96–106).

However, similar to any model, humanized mice have their practical limitations. For example, they may not completely replicate the entire immune system, potentially resulting in suboptimal functionality of components like B and NK cells. Nevertheless, researchers have the opportunity to explore specific protocol adjustments to promote the maturation of specific cell populations in these mice (107–109). Another drawback pertains to the potentially high cost for researchers, especially if they opt to purchase commercially available humanized animals. Additionally, experiments may be constrained by the shipping and engraftment schedules of the companies, and the humanization status will not be retained if these animals reproduce. Moreover, when attempting in-house humanization, graft-rejection rates can be quite high, as the innate murine immune system, albeit diminished, remains present (110). In some robust transgenic models and in the transiently humanized mice, acquired innate immune response from the HSC may reject some PDX models if there is a Major histocompatibility complex (MHC)-mismatch between the engrafted HSC and the PDX. This is precisely why these models are particularly valuable for studying graft-versus-host disease in the transplantation (75, 92, 110–116). However, as previously described, transiently humanized mice may allow the use of specific HSCs that are histocompatible with the PDX models, enabling the incorporation of patient-derived cells or tissues, facilitating personalized medicine (79, 114). On the other hand, models of humanized mouse models lack the ability to process and present antigens efficiently, primarily due to deficient B cells in responding to foreign antigens. This is crucial for mounting an effective defense against infections (75, 117, 118).

## Humanized mice and chordoma research

### Tumor (immune) microenvironment to motivate choice for immunocompetent vs immunocompromised mice

Tumor cells are not isolated entities; instead, they coexist within a complex environment made up of various cell types, extracellular matrix, and other molecular components. A tumor isn’t solely a cluster of cancer cells; it represents a heterogeneous collection of many different types of resident and infiltrating cells, secreted factors and the ECM. This complex entity is commonly referred as the tumor microenvironment (TME) (119–121). It has been suggested that the TME is not just a silent bystander, but plays a crucial role in cancer progression, either promoting or inhibiting cancer growth and metastasis depending on its composition (121).

Immune cells are a major component of the TME and its presence and activity can greatly influence cancer progression. Tumor-infiltrating lymphocytes (TILs), such as cytotoxic T cells, and Natural Killer (NK) cells can recognize and attack the cancer cells. However, tumor cells have different mechanisms to evade the immune system, such as immune checkpoints, that allows cancer to thrive (122). Understanding the complexity and the mechanisms for which the tumor immune microenvironment (TIME) affects the tumor may unveil immunotherapies (123–125). The TIME refers to the different subpopulations of the immune system that may infiltrate the tumor at a certain time and that may have different effects in tumor initiation, progression and response to therapies (124, 126, 127).

### Use of humanized mice in chordoma research

While immunocompromised mice have been valuable options for *in vivo* cancer research, emerging case studies, cellular data, and clinical trial findings related to the immunological alterations observed in chordoma, as well as the potential chordoma therapies that leverage the immune system, underscore the significance of possessing a representative model of the human immune system. Such a model is essential for comprehensively characterizing the underlying mechanisms involved in chordoma and its potential therapeutic strategies (128–135). As previously discussed, TME plays a crucial role in tumor progression, and this is particularly true for the TIME, which can significantly influence the malignancy of tumor cells, including chordoma. For instance, tumor-associated macrophages, which are among the most prevalent cell types within the TIME and infiltrate the chordoma tumor parenchyma, are highly versatile macrophages capable of transitioning between M1 and M2 phenotypes depending on the specific requirements of the tumor (136). Therefore, it has been suggested that one of the ways to target cancer is to enhance immune regulation (137, 138). For example, the programmed death-1 (PD-1)/programmed death ligand-1 (PD-L1) pathway has been widely studied by Zou and colleagues (35, 62, 128, 139, 140) as TILs and macrophages express PD-1 receptor (138). Besides the PD1/PDL1 pathway, TGF-beta-related genes, HHLA2, and cytotoxic T-lymphocyte antigen-4 (CTLA-4) are among some of the immune-associated molecules analyzed so far (128, 129, 131). Other examples of interactions between chordoma and the immune system are the above mentioned plastic macrophages that, via the ccl5/ccr5 axis, are recruited and polarized by chordoma to enhance the proliferation, invasion and migration capabilities of chordoma (136); galectin-9 that interacts with TIM3-positive TILs and that promotes cell apoptosis (141) and the expression of CTLA-4, a promising immune checkpoint inhibitor target that it is expressed by the TILs that are infiltrating chordoma (129). We can learn from these examples that chordoma can evade the immune system by evading the recognition via antigen restriction by the TIL and marrow-infiltrating lymphocytes (MIL), or by directly interacting with the immune system and inhibiting it or by causing T cell exhaustion, as happens in many other cancers (142). Moreover, not only the TIME can regulate immune escape, it has been suggested in other cancers that can also promote distant metastasis and the acquired resistance to chemotherapy and radiotherapy (143–145). CAR-modified T cells that could inhibit the molecule B7-H3 are also being explored in chordoma (146). Various types of humanized mice have been used to assess therapies for other cancers and could potentially be applied to chordoma as well (147).

As we discussed above, most of the immunocompromised mice lack one or more cells from the immune system so the host doesn’t reject the human cell/graft. For example, NU/J athymic nude mice lack T cells and that partially affects B cells maturation. However, understanding the complex interactions between the immune system, the TME, the TIME and the tumor itself is crucial for, not only the development of new immunotherapies, but also takes into account the effect of these interactions in cell transformation and resistance to therapies. Humanized mice are then valuable tools for studying cancer and the interactions between the human immune system and tumor cells. These mice are engineered to generate functional human immune cells, enabling the research of the complex dynamics of the human immune response within a living organism, providing a bridge between *in vitro* studies and clinical trials. Also, they provide a system that is ideal for targeting immunotherapies that involve T and B cells responses. Also, it is a system that is ideal for the PDXs as it more closely recapitulates the tumor heterogeneity and the TIME interactions, being the most predictive preclinical oncological model.

Even though immunocompromised mice have served to obtain an immense amount of knowledge on basic biology, there are limitations on the use to study human biology and disease. The first of all is that the host innate immune system of a mouse is different from the immune system of a human. That’s why many drugs and pathogens are species-specific (148). Immunocompromised mice are leading to the implementation of personalized medicine: engraftment of mice with patient-derived tumors allows researchers to find and evaluate specific therapies that can ultimately help the patient. Humanized mice not only allow the study of immunotherapies that depend on the engrafted immune system, it also gives the researcher context on the response of the tumor cells and the TIME against any other chemotherapeutic drug and even combination of drugs (149). Moreover, they are a good model on investigating mechanisms of tumor metastasis and to study the immune checkpoint mechanisms (69, 149). It has also been demonstrated that the effect of the TME and the TIME may lead to intra-tumoral heterogeneity and differential chordoma classification that may lead to differential prognosis and response to treatment (150–155).

Previously, altered expression of some genes was found to be associated with chordoma, and molecular-based treatments may mitigate the downstream changes instigated by this genetic variability (34). *In vivo* cancer models, as compared to *in vitro* studies, may more accurately reflect dynamic molecular changes, including genetic expression, of tumors (156), and these models are therefore a crucial component to the research process. Prior attempts to grow chordoma in immunocompromised mice produced tumors that were largely histologically and genetically akin to the original patient-derived cells. However, with researchers exploring immunotherapy as an additional route of chordoma treatment, an equally effective *in vivo* model preserving immune function is needed to determine efficacy.

Lim et al. (2015) noted this limitation. They identified PD-1 expression in chordoma immune cells *in vitro*, potentially implicating this molecule’s interaction with PD-L1 receptors on chordoma cells in cancer cell survival (157). A partnership with Jackson Laboratories led to novel development of a humanized mouse for chordoma in 2015 (156). A team of researchers, which included those involved in the creation of this mouse, initiated a study calling for the need to investigate chordomas through such mice, wherein the immune system would be compatible with the human chordoma cells (158). They then utilized a humanized mouse to evaluate the efficacy of potential chordoma treatments. Interestingly, the study identified that treatments against PD-1 adjuvant to radiation resulted in relatively few cytotoxic T cells expressing PD-1 and high levels of several other types of human immune cells alongside a reduced tumor size (159). Since immunodeficient mice do not possess these other immune cells, these cells’ involvement in tumor suppression following immunotherapy—such as in this context of attenuated PD-1 and PD-L1 binding—may be insufficiently studied without a humanized mouse model. As we already mentioned, Xu et al. (136) found that ccl5 and M2 macrophages drive chordoma growth, and while they acquired this data through use of an immunodeficient mouse, the lack of a humanized mouse limited the team’s ability to assess the underlying molecular characteristics. Another research team from Northwestern University has also proposed a project to create a humanized mouse for the study of chordoma therapies (160). Other research has further elucidated potential immunotherapies for chordoma, and a humanized mouse model may enable more extensive *in vivo* studies of these therapy candidates. Gounder et al. (2019) showed through clinical trial data that impeding EZH2 activity in a chordoma patient extended the expected survival while changing the immune composition of the tumor (161). Hoke et al. (2019) and Fujii et al. (2020) enhanced natural killer cell effects *in vitro* by targeting the epidermal growth factor receptor and other ligands and cytokines to activate antibody-dependent cell-mediated cytotoxicity (ADCC) (162, 163).

Chordoma possesses diverse genetic, epigenetic, and immunotherapeutic targets that may be a target for combinatorial therapies (164), which will need the appropriate models, from *in vitro* to the different animal models to test those therapies. In summary, immunocompromised mice are useful for studying tumor growth and responses to treatment independently of the immune system interference, while humanized mice provide a platform to study the interactions between human tumors and the human tumor immune microenvironment (Summarized in **Table 2**). The choice between the two models, and the different array of models within each one, will depend on the research questions being asked.

**Table 2:** A comparison of the applications of immunocompromised and humanized mouse models in cancer and tumor microenvironment studies. Created with BioRender.com.

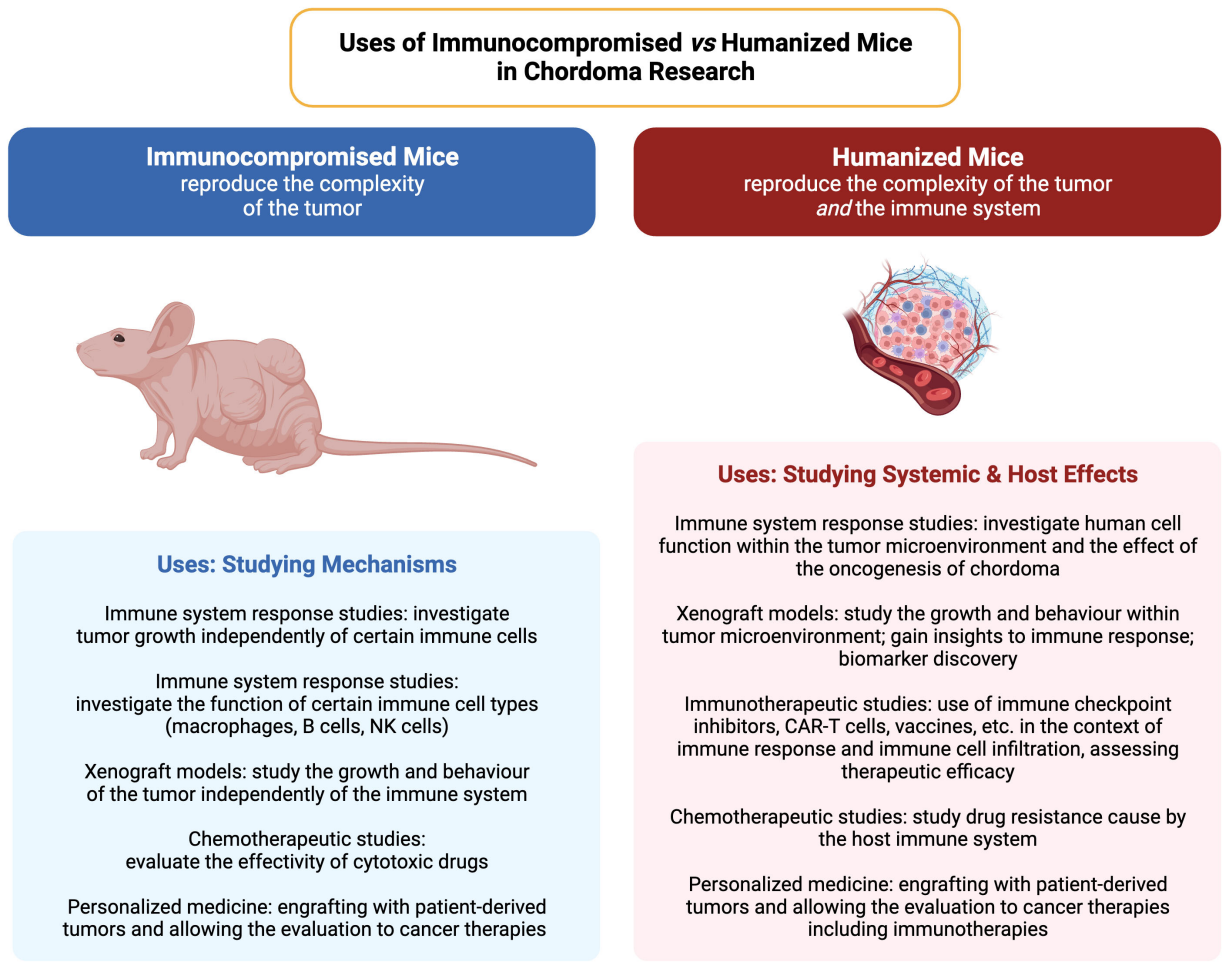

## Conclusions and future directions

Humanized mice models provide a platform with which to investigate different cancers in the context of the tumor microenvironment and the field of immuno-oncology (75, 115, 165). These models are generally generated by the engraftment of HSCs or PMBCs into immunocompromised mice to mimic the human immune system and are very useful for the implantation of human cancer CDXs and PDXs. Even though some humanized mice approaches have been made in relation to chordoma, there is a wide array of possibilities that can be applied to chordoma research to find therapies. In conclusion, there is rationale for the use of humanized mice to study chordoma in the context of the TME.

## Author contributions

BC: Conceptualization, Investigation, Writing – original draft, Writing – review & editing. CS: Conceptualization, Investigation, Writing – original draft, Writing – review & editing. ES: Conceptualization, Investigation, Writing – original draft, Writing – review & editing. JA: Investigation, Writing – original draft, Writing – review & editing. OL: Investigation, Writing – original draft, Writing – review & editing. ZG: Writing – review & editing, Supervision. PS: Supervision, Writing – review & editing, Conceptualization. MM-M: Conceptualization, Supervision, Writing – review & editing, Formal analysis, Investigation, Writing – original draft.
